# Comparative transcriptome analysis revealed that auxin and cell wall biosynthesis play important roles in the formation of hollow hearts in cucumber

**DOI:** 10.1186/s12864-024-09957-x

**Published:** 2024-01-05

**Authors:** Jiaxi Li, Chenran Gu, Yanwen Yuan, Zeyuan Gao, Zhiwei Qin, Ming Xin

**Affiliations:** Key Laboratory of Biology and Genetic Improvement of Horticultural Crops (Northeast Region), College of Horticulture and Landscape Architecture, Northeast Agricultural University, 150030 Harbin, Heilongjiang China

**Keywords:** Cucumber, Hollow heart, Comparative transcriptome analysis, Auxin, Cell wall

## Abstract

**Background:**

Hollow heart is a kind of physiological defect that seriously affects the yield, quality, and economic value of cucumber. However, the formation of hollow hearts may relate to multiple factors in cucumber, and it is necessary to conduct analysis.

**Results:**

In this study, hollow and non-hollow fruits of cucumber K07 were used for comparative transcriptome sequencing and analysis. 253 differentially expressed genes and 139 transcription factors were identified as being associated with the formation of hollow hearts. Hormone (auxin) signaling and cell wall biosynthesis were mainly enriched in GO and KEGG pathways. Expression levels of key genes involved in indole-3-acetic acid biosynthesis in carpel were lower in the hollow fruits than non-hollow fruits, while there was no difference in the flesh. The concentration of indole-3-acetic also showed lower in the carpel than flesh. The biosynthetic pathway and content analysis of the main components of the cell wall found that lignin biosynthesis had obvious regularity with hollow heart, followed by hemicellulose and cellulose. Correlation analysis showed that there may be an interaction between auxin and cell wall biosynthesis, and they collectively participate in the formation of hollow hearts in cucumber. Among the differentially expressed transcription factors, MYB members were the most abundant, followed by NAC, ERF, and bHLH.

**Conclusions:**

The results and analyses showed that the low content of auxin in the carpel affected the activity of enzymes related to cell wall biosynthesis at the early stage of fruit development, resulting in incomplete development of carpel cells, thus forming a hollow heart in cucumber. Some transcription factors may play regulatory roles in this progress. The results may enrich the theory of the formation of hollow hearts and provide a basis for future research.

**Supplementary Information:**

The online version contains supplementary material available at 10.1186/s12864-024-09957-x.

## Background

Cucumber (*Cucumis sativus* L.) is an important vegetable consumed worldwide. Hollow heart, also called internal cracking or fruit cavity, is usually manifested as the crack in the center of the fruit but the appearance is normal. Hollow heart is a kind of physiological defect that seriously affects the yield, quality, and economic value of cucumber. The formation of hollow heart was related to multiple growth conditions and genetic characteristics and the reason may not be the same in different species.

The formation of hollow heart may be affected by the cultivation environment, in which water is a major element. When potatoes were short of water, the content of soluble sugar increased in the tubers and led to a decrease in water potential. The cells around the pith grow faster than those in the intramedullary space, thus forming hollow heart [1]. Similarly, in radishes, improper irrigation led to hollow and malformed roots [2]. Temperature is another key factor causing the hollow heart of fruits. When peas grow at 33 ℃ (daily average temperature) for 5 days, 80% of the seeds will appear hollow [3]. Long-term (more than 12 days) low temperature led to a hollow heart in watermelon. The reason was that the low temperature slowed down the cell division rate after fruit set, and the peel grew rapidly when the temperature increased in the later stage, so the number of cells in the fruit was insufficient [4]. Hollow hearts were also suspected to be related to mineral elements in previous studies. Localized tissue calcium deficiencies were implicated as mechanisms initializing cell death and tissue necrosis leading to hollow hearts in potato [5]. In citrus, if the supply of phosphorus was insufficient, the possibility of hollowness in the later stage of fruit development would increase [6]. Unseemly application of nitrogen fertilizer will also lead to an increase in the rate of hollow hearts before or during the potato tuber formation period [7]. In addition, harvest time [8], pests or diseases [9, 10], and cultivation methods [11, 12] may also cause hollow hearts.

Researchers have explored the genetic and molecular mechanism of hollow hearts from many aspects. John et al. detected a quantitative trait locus (QTL) associated with internal growth cracks in potato on chromosome 5 by using genetic populations and molecular markers [13]. Later, five QTLs were identified for the incidence of hollow heart in potato [14]. Similarly, a QTL related to hollow steam was identified in broccoli [15]. These QTLs can be further studied and develop markers for marker-assisted selection. Combined fine-mapping and omics analyses, a structural variant in the promoter of the *AFF* (all-flesh fruit) gene was identified to be related to the hollow heart of tomato fruit. The formation of hollow heart was due to the ventricle could not be filled after the ovary tissue changed from gel-like material to solid tissue [16]. Transcriptome analysis suggested that the juice sac granulation was closely related to cell wall metabolism, and low temperature would aggravate the granulation in late-ripening navel orange (*Citrus sinensis* Osbeck) [17]. Transcriptome analysis also revealed that abscisic acid might inhibit the synthesis of indole-3-acetic acid (IAA), zeatin riboside, and gibberellins and cause fruit cavity formation in Nai plum (*Prunus salicina*) [18].

Some studies have tried to explain the formation of hollow heart in cucumber. Hollow size (carpel rupture) was found to be controlled by a single gene in immature cucumber fruit [19] and by two or three genes at the mature stage [20]. Five QTLs were detected in the recombinant inbred lines, and two were detected in the F_2:3_ population for hollow size using Sikkim cucumber (*Cucumis sativus* var. *Sikkimensis*) for the first time [21]. Among the QTLs, two (*mfh2.1* and *mfth3.1*) were identical in both populations, proposing that they may be closely related to the hollow heart of cucumber [21]. The hollow heart of cucumber fruit was found to be consistent with the 3:1 segregation ratio in the F_2_ population of South China ecotype cucumber, suggesting that the trait was regulated by a single dominant gene. Then, aluminum-activated malate transporter 2 (*CsALMT2*) was identified based on bulked segregation analysis and kompetitive allele-specific PCR (polymerase chain reaction) analysis [22]. With the development of technology, an increasing amount of research will tend to be carried out from the perspective of gene regulation. *CsGID1a*, a gibberellin receptor gene, was found to be closely related to locule formation in cucumber fruit. Silencing of *CsGID1a* led to abnormal development of carpels ventricles and hollow hearts but did not affect seed development [23]. Cheng et al. found that *SPATULA* and *ALCATRAZ* (encoding two bHLH transcription factors) act in homodimers and heterodimers to confer female sterility and fruit cavity by mediating genes involved in transmitting tract development in cucumber [24]. It can be seen that the formation of hollow heart may relate to multiple pathways in cucumber.

Due to the diversity and complexity of influencing factors in the formation of hollow heart, it is necessary to conduct analysis. Based on the investigation and screening of the hollow heart of cucumber germplasm resources in a previous study [25], transcriptome sequencing and analysis were performed on both hollow and non-hollow cucumbers. This study was undertaken to (i) identify the differentially expressed genes and transcription factors, (ii) identify the biological pathways associated with the formation of hollow hearts, and (iii) predict possible regulatory patterns. The results may enrich the theory of the formation of hollow heart and provide a basis for future research.

## Results

### Phenotype identification

**Fig. 1:**
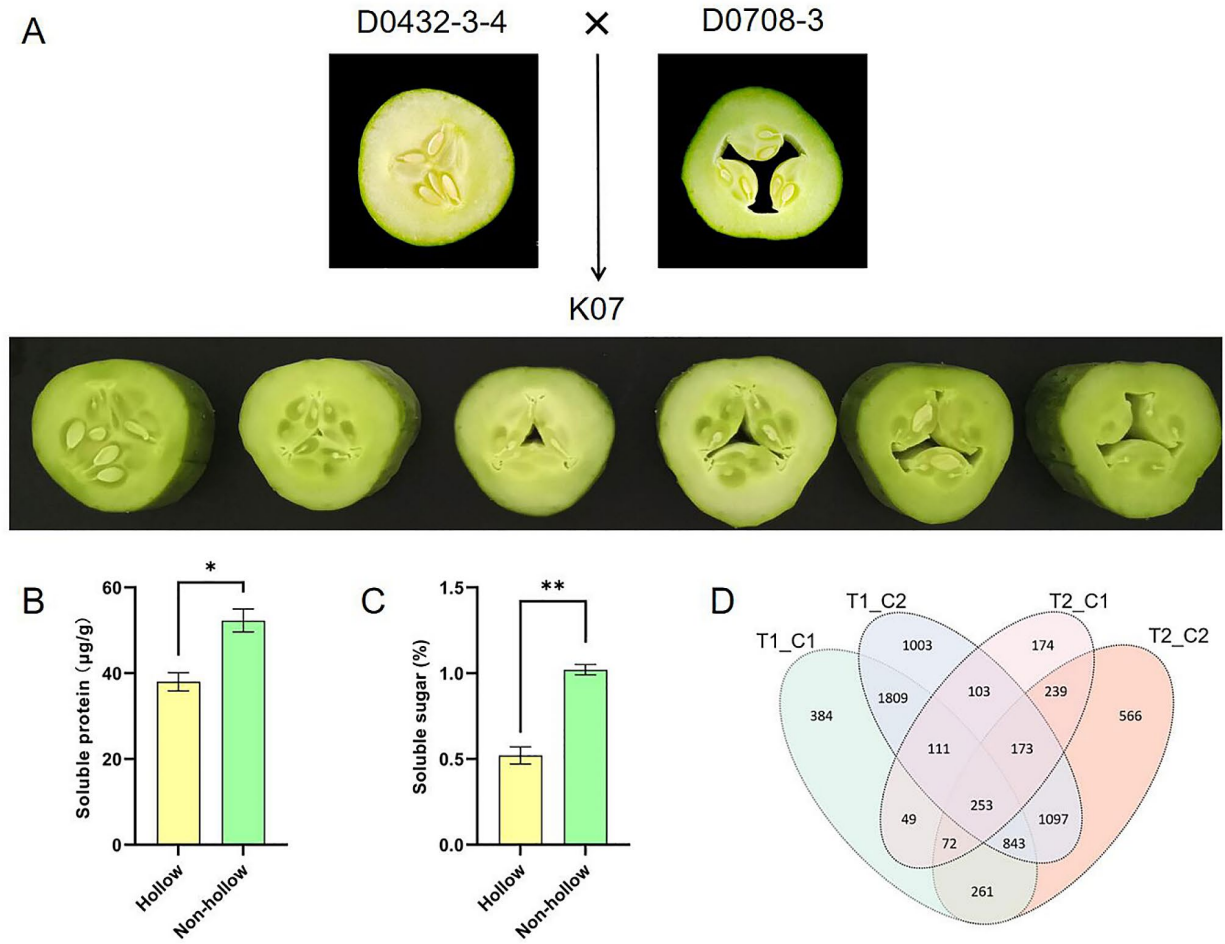
Phenotype, concentration of soluble protein and soluble sugar, and differentially expressed genes of hollow and non-hollow cucumber fruits. (**A**) Cross-sections of D0432-3-4 (♀), D0708-3(♂), and K07 (F_1_) cucumber fruit. The six cross-sections of K07 showed non-hollow and different degrees of hollow hearts; (**B** and **C**) Concentration of soluble protein and soluble sugar in hollow and non-hollow cucumber fruit, respectively; (**D**) Venn diagram of differentially expressed genes in four comparison groups for transcriptome sequencing. Asterisk symbols (*, **) represented the significant results at **p < 0.01 and *p < 0.05 level, respectively

Under the same growing environment and field management methods, the cross-sections of fruits on the 9th day after pollination were showed (Fig. 1A). Fruits of D0432-3-4 showed non-hollow hearts while fruits of D0708-3 showed hollow hearts. Fruits of K07 showed non-hollow and different degrees of hollow hearts on a plant. The hollow degree varied from 3.7 to 20.5%. Hollower heart may be caused by some genetic factors, and the transcriptome analysis was followed to explore the mechanism.

There was a significant difference in concentration of soluble protein between the hollow fruit and the non-hollow fruit, and the hollow fruit was reduced by approximately 25% (Fig. 1B). There was an extremely significant difference in concentration of soluble sugar between hollow fruit and non-hollow fruit, and the hollow fruit decreased by approximately 50% (Fig. 1C). The results show that the hollow heart not only affected the commodity quality of cucumber but also had a great influence on the nutritional quality.

### Transcriptome sequencing and differentially expressed gene (DEG) analysis

A total of 508,600,056 clean high-quality reads (more than 90% of raw reads) were obtained from 12 cucumber samples after removing polluted reads, low-quality reads and Ns reads. The sequencing data were uniform in the whole genome coverage. The clean Q30 base rate was more than 93%. The mapping rate was more than 95%, and 88% of the sequences were mapped to exons. The occurrence frequency of the four bases was almost the same and there was no phenomenon of location difference and AT/GC separation. The sequencing data quality control was qualified and can be used for further analysis.

Four comparison groups (T1_C1, T1_C2, T2_C1, and T2_C2) were constructed to obtain the DEGs in hollow and non-hollow heart fruits of cucumber. A total of 7,137 DEGs were identified after mapping to the cucumber (Chinese Long) v3 genome [26] and were identified in the four groups (Fig. 1D). Among these comparison groups, the T2_C1 group showed the largest difference in gene expression, with 5,392 DEGs, while there were only 1,174 DEGs in the T2_C2 group. Compared with flesh, there were more differentially expressed genes in the carpel, indicating that the hollow heart may be related to the development of the carpel. A total of 253 DEGs appeared in all groups in the Venn diagram, which may be closely related to the formation of hollow heart (Additional File 2).

### Functional enrichment analysis of DEGs

**Fig. 2:**
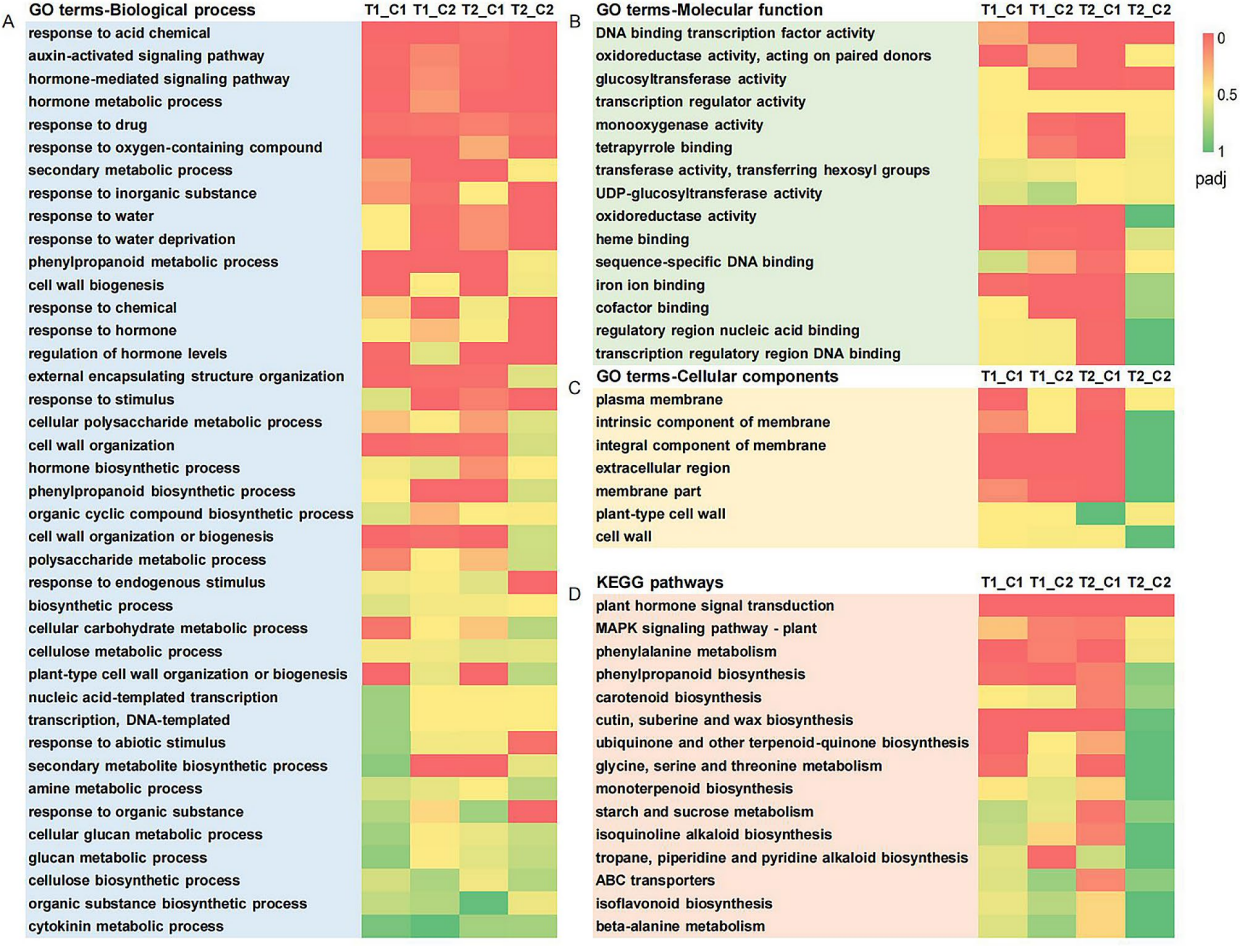
GO and KEGG pathways functional enrichment analysis of DEGs. (**A**) Enriched biological process pathways of GO; (**B**) Enriched molecular function pathways of GO; (**C**) Enriched cellular components pathways of GO; (**D**) Enriched KEGG pathways. Padj, the corrected *p* value

DEGs of T1_C1, T1_C2, T2_C1, and T2_C2 were enriched into 5332, 4991, 6243, and 3270 Gene Ontology (GO) entries, respectively. The mainly enriched GO terms in each category were further analyzed by combining the four comparison groups. In terms of biological processes, most of the forty enriched terms were related to hormone signaling (auxin and cytokinin), cell wall biosynthesis or metabolism, polysaccharide (cellulose) metabolism, and organic substance (cyclic compound, especially phenylpropanoid) biosynthetic processes. Some terms of response to environmental conditions and chemical substances were also significantly enriched (Fig. 2A). DNA binding transcription factor activity, oxidoreductase activity and glucosyltransferase activity were located in the top three of fifty mainly enriched terms in the category of molecular function (Fig. 2B). Seven terms were enriched in the cellular components and were mainly related to the cell membrane, cell wall and extracellular region (Fig. 2C).

Kyoto Encyclopedia of Genes and Genomes [27] (KEGG) pathway enrichment analysis was performed on the combination of enrichment pathways for all four comparison groups (Fig. 2D). The results showed that DEGs were mainly significantly enriched in plant hormone signal transduction (278 DEGs), MAPK (mitogen-activated protein kinase) signaling pathway-plant (137 DEGs) and phenylalanine biosynthesis (145 DEGs) or metabolism (46 DEGs). Combining the results of GO with KEGG enrichment, the hollow heart of cucumber may be closely related to hormone signaling and cell wall biosynthesis.

### Gene expression patterns of the auxin biosynthesis pathway

**Fig. 3:**
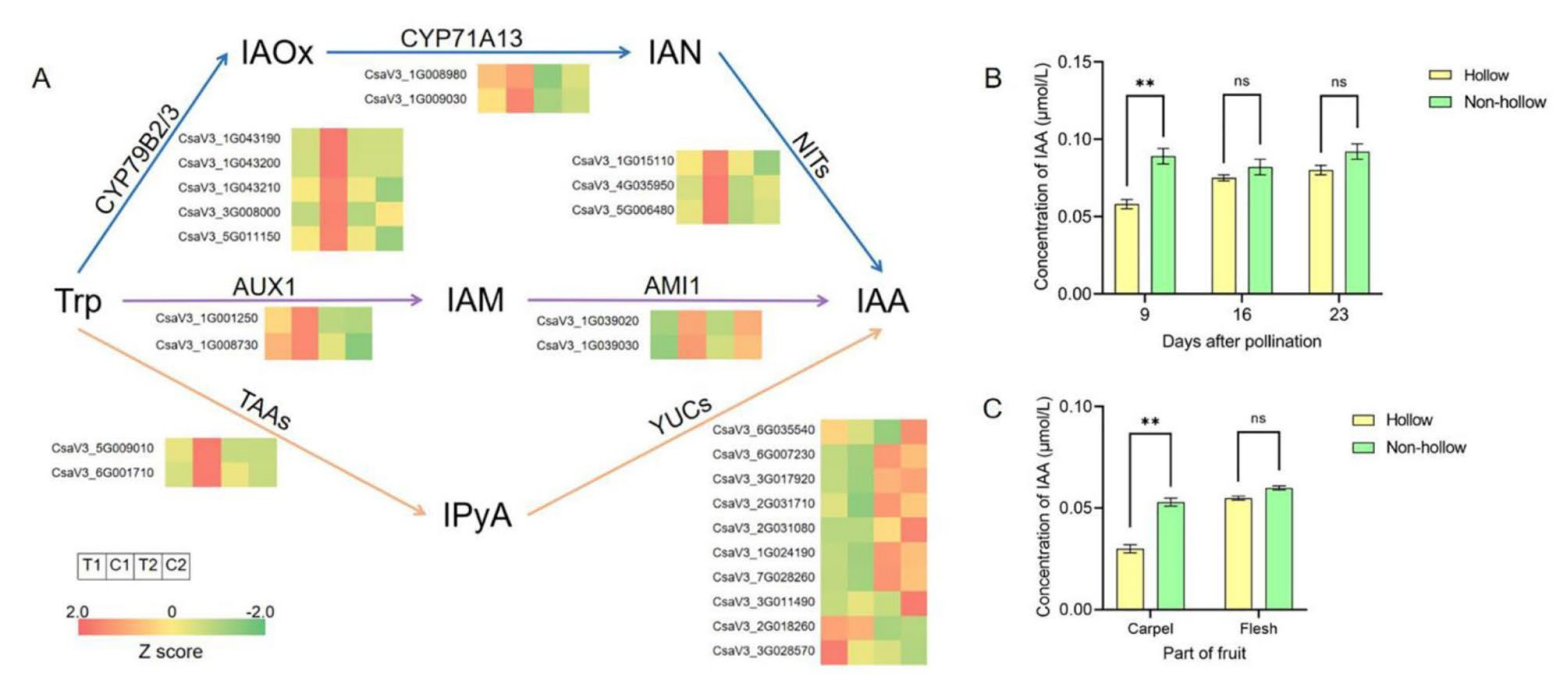
Expression profiles of genes involved in the biosynthesis pathways of IAA and concentration of IAA in hollow and non-hollow fruits. (**A**) Expression profiles of genes involved in biosynthesis pathways of IAA. IAN, indole-3-acetonitrile; (**B**) Concentration of IAA in the middle (including fruit peel, flesh, and carpel) of hollow and non-hollow fruits on the 9th, 16th, and 23rd days after pollination; (**C**) Concentration of IAA in carpel and flesh of hollow and non-hollow fruits on the 9th day after pollination. Asterisk symbol (**) represented the significant results at **p < 0.01 level. Ns, no significant difference

Auxin biosynthesis pathways include tryptophan- and nontryptophan-dependent pathways, and the tryptophan-dependent biosynthesis pathway is the main pathway for IAA, which is the most studied auxin in plants [28]. Tryptophan aminotransferase of Arabidopsis (TAA), catalyzing tryptophan (Trp) to indole-3-pyruvic acid (IPyA), is the key enzyme in the IPyA pathway of IAA biosynthesis. According to the expression profile, the expression levels of *TAAs* were lower in the carpel of hollow cucumber fruit than non-hollow fruit, while there was no difference in the flesh. The expression levels of *YUCCA* (*YUCs*) in the carpel were lower than flesh. but they were similar between hollow and no-hollow fruits. Compared with other *YUCs*, *CsaV3_028570* showed a special expression profile, which may participate in other biological processes and perform different functions in the formation of hollow heart (Fig. 3A). In the indoe-3-acetaldoxime (IAOx) pathway, the expression levels of cytochrome P450 monooxygenase (*CYP79Bs* and *CYP71A13*) and nitrilase (*NITs*) were all lower in carpel of hollow fruit than non-hollow fruit, while there was no difference in flesh (Fig. 3A). In the indoleacetamine (IAM) pathway, the expression patterns of auxin 1 (*AUX1*) and amidase 1 (*AMI1*) were similar to the two pathways described above (Fig. 3A).

On the 9th day after pollination, the time at which the transcriptome sequencing sample was obtained, the concentration of IAA in the hollow fruits was about 35% lower than that in the non-hollow fruits, which was consistent with the expression patterns of genes involved in the biosynthesis of IAA. But there was no significant difference between the two on the 16th and 23rd days after pollination (Fig. 3B). The IAA concentration in hollow fruits gradually increased, while that in non-hollow fruits was little change and always higher than hollow fruits, and both reached a maximum on the 23rd day. Furthermore, the concentration of IAA in the carpel and flesh was determined on the 9th day after pollination, respectively. The concentration of IAA in carpel showed significant difference between hollow and non-hollow fruits, while there was no difference in flesh (Fig. 3C). According to the above analysis, the low expression levels of these key genes in the IAA biosynthetic pathway affected the concentration of auxin in the carpel of fruit at the early stage of development, which may be the one of the reasons causing hollow heart of cucumber fruit.

### Gene expression patterns of the cell wall biosynthesis pathways

In GO and KEGG enrichment, cell wall biosynthesis pathways were significantly enriched (Fig. 2). The plant cell wall includes the primary wall and secondary wall, which are composed of different proportions of cellulose, hemicellulose, lignin, pectin, and some wall proteins [29]. Here, the expression patterns of three main components (lignin, cellulose, and hemicellulose) of the cell wall were analyzed.

**Fig. 4:**
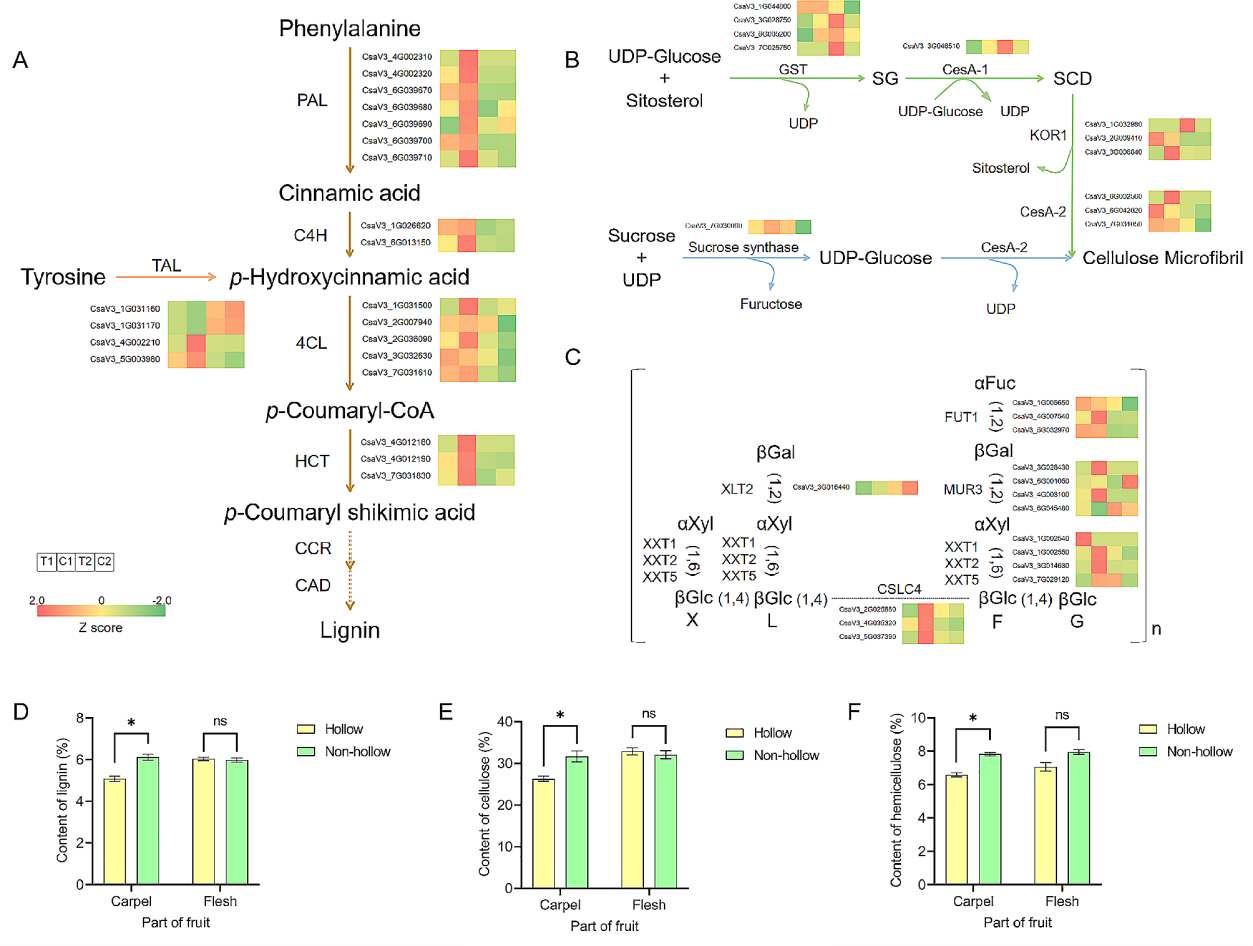
Expression profiles of genes involved in biosynthesis pathways and the content of lignin, cellulose, and hemicellulose (represented by XyG). (A, B, and C) Genes expression profiles in the biosynthesis pathways of lignin, cellulose, and XyG (XLFG structure), respectively.; (D, E, and F) Content of lignin, cellulose, and hemicellulose in carpel and flesh of hollow and non-hollow fruits on the 9th day after pollination, respectively. Asterisk symbol (*) represented the significant results at *p < 0.05 level. Ns, no significant difference. CCR, cinnamoyl-CoA reductase; CAD, cinnamyl-alcohol dehydrogenase; UDP, uridine diphosphate; GST, Glucose sterol glucosyltransferase; SG, sitosterol-glucoside; CESA, cellulose biosynthesis; SCD, synthesizes sitosterol cellodextrin; KOR1, glucanase; αFuc, αFucose; βGal, βGalactoseα; Xyl, Xylose; XLT2, XyG L-side chain galactosyltransferase

The expression levels of the key genes (including phenylalanine ammonia lyase, *PAL*; cinnamic acid 4-hydroxylase, *C4H*; 4-coumarate-CoA ligase, *4CL*; hydroxycinnamoyl transferase, *HCT*) in the lignin biosynthesis pathway were higher in the carpel of non-hollow fruit than hollow fruits, but there was no significant difference in the flesh. The expression pattern of four *TAL* (tyrosine ammonia lyase) genes was not consistent (Fig. 4A), indicating that the biosynthesis from *p*-hydroxycinnamic acid to tyrosine may not closely relate to hollow heart. It was speculated that low expression levels of genes in the biosynthesis pathway of lignin (especially from phenylalanine to *p*-coumaryl shikimic acid) affected its content in the carpel of fruit. Cellulose in plants mainly exists in the form of microfibrils, which are generally crystallized from 36 β-1,4-glucoside chains [30]. In the biosynthesis pathway of cellulose, there was no regularity between the expression pattern of related genes and the hollow heart (Fig. 4B), indicating that there was little relevance between cellulose and the formation of hollow heart. Xyloglucan (XyG) is the most abundant hemicellulose in the primary wall of dicotyledonous plants. The main sugar chain is connected by a β-(1, 4)-glycosidic (βGlc) bond and the branch chain is replaced by a variety of glycosyl and ferulic acid groups [31]. In the biosynthesis pathway of XyG (XLFG structure), glucan synthase 4 (*CSCL4*) also showed higher expression levels in the carpel of non-hollow fruits than hollow fruits, but there was no difference in the flesh. Some homologs of fucosyltransferase (*FUT1*), *MUR3* (encodes a xyloglucan galactosyltransferase that acts specifically on the third xylose residue within the core structure of XyG [32]) and xyloglucan xylosyltransferases (*XTTs*) also showed similar expression patterns (Fig. 4C). However, most of homologs showed no regularity between the expression pattern and hollow heart.

On the 9th day after pollination, the content of lignin in the carpel of hollow fruits was 27% lower than non-hollow fruits (Fig. 4D), which was consistent with the expression patterns in the biosynthesis pathway. The content of cellulose and hemicellulose were also lower in the carpel of hollow fruits than non-hollow fruits, but there were no significant difference in the flesh (Fig. 4E&F). According to the above analysis, it was speculated the low expression levels and low contents of lignin, cellulose, and hemicellulose affected the synthesis of cell wall and caused hollow hearts.

### Correlation of genes between IAA and cell wall biosynthesis

**Fig. 5:**
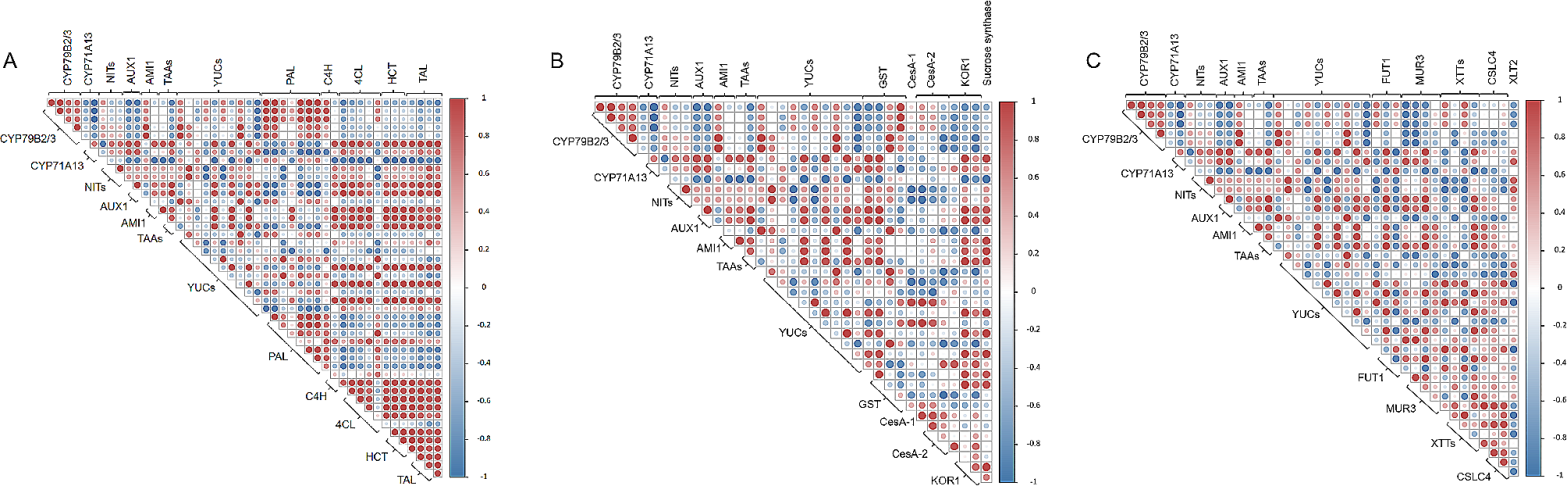
Pearson coefficient matrix analysis between genes involved in IAA and cell wall biosynthesis. Corrplot between genes in the biosynthesis pathways of IAA and lignin (A), IAA and cellulose (B), IAA and XyG (C)

The above analysis found that IAA and cell wall biosynthesis pathways may be related to the formation of hollow heart. Here, Pearson coefficient matrix analysis was performed to explore whether there was a correlation between these biosynthesis pathways. Among the genes in the biosynthesis pathways of IAA and lignin, *CYP79B2/3* was mainly positively correlated with *PAL*. *CYP71A13* was negatively correlated with *PAL* and positively correlated with *4CL*, *HCT*, and *TAL*. *AUX1*, *AMI1*, and *TAAs* positively correlated with *C4H*, *4CL*, *HCT*, and *TAL*. *NITs* and *YUCs* were weakly correlated with genes in the biosynthesis pathways of lignin (Fig. 5A). For genes in biosynthesis pathways of IAA and cellulose, *CYP79B2/3* was mainly negatively correlated with *KOR1*, and *NITs* were mainly negatively correlated with *CseA*. *CYP71A13*, *AUX1*, *AMI1*, and *TAAs* were mainly positively correlated with genes in the biosynthesis pathway of cellulose (Fig. 5B). Among the genes in the biosynthesis pathways of IAA and XyG, *CYP79B2/3* was mainly negatively correlated with *MUR3* while *AUX1* showed positively correlated with *MUR3*. *NITs* showed negative correlation with genes in the biosynthesis pathways of XyG. There was no obvious regularity among other genes (Fig. 5C). Results showed that there may be interaction between IAA and cell wall biosynthesis, and they collectively participate in the formation of hollow heart in cucumber.

### Analysis of differentially expressed transcription factors (TFs)

**Fig. 6:**
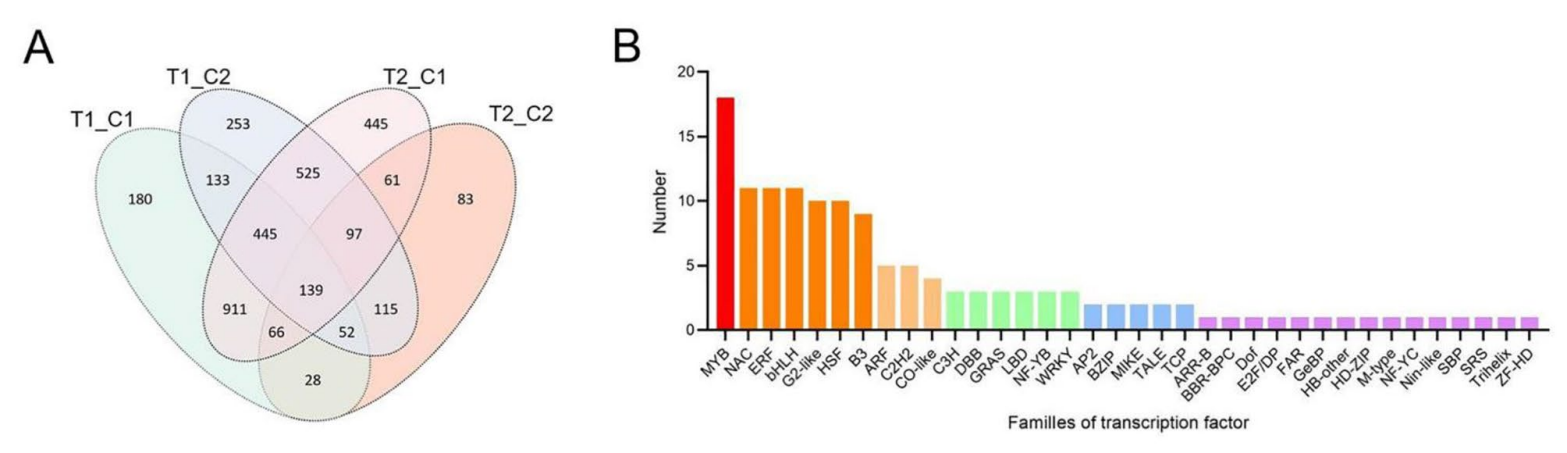
Analysis of differentially expressed TFs in transcriptome sequencing. (**A**) Venn diagram of differentially expressed TFs between four comparison groups; (**B**) Annotation of the families of 139 common differentially expressed TFs in four comparison groups

A total of 3,533 TFs were identified after mapping to the cucumber (Chinese Long) v3 genome [26]. Among these comparison groups, the T2_C1 group showed the largest difference in gene expression, with 2,689 TFs, while there were only 641 TFs in the T2_C2 group (Fig. 6A). A total of 139 TFs appeared in all groups and may be closely related to the formation of hollow hearts (Additional File 3). Furthermore, the 139 TFs were annotated to 36 families. The v-myb avian myeloblastosis viral oncogene homolog (MYB) family has the most members (18). It was worth noting that six MYB-related genes were annotated as xyloglucan endotransglucosylase/hydrolase 16, which involved in the biosynthesis of XyG. Among them, *CsaV3_3G036070* changed most significantly with downregulated expression (Log2FoldChange = 8.31) in the T1_C1 group (Additional File 3). The members of the NAC (including NAM, ATAF1/2, and CUC1/2), ethylene response factor (ERF), basic helix-loop-helix (bHLH), Golden2-like (G2-like), and heat shock factor (HSF) families ranged from 8 to 11. There were five members of auxin response factor (ARF) and Cys2/His2 (C2H2). The remaining families were annotated to fewer than 5 members (Fig. 6B). Transcription factors play important roles in the formation of plant traits, and the annotated differentially expressed TFs may provide support for further research.

### Quantitative real-time PCR (qRT‒PCR) verification

**Fig. 7:**
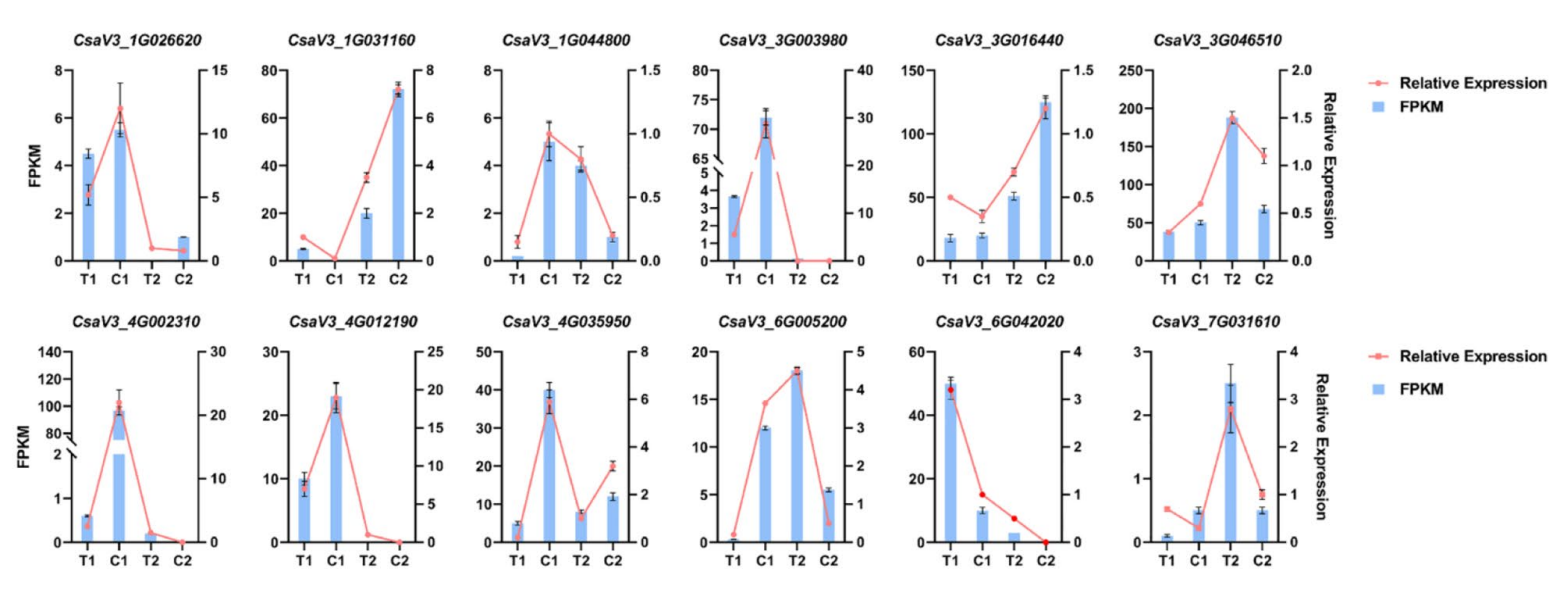
Quantitative verification of twelve genes in the biosynthesis pathway of IAA and the cell wall

Twelve DEGs selected from the biosynthesis pathway of IAA and cell wall were used for qRT‒PCR verification. The relative expression trends of twelve DEGs were consistent with the fragments per kilobase of exon model per million mapped fragments (FPKM) obtained by transcriptome sequencing, indicating that transcriptome sequencing data were reliable in this study (Fig. 7).

## Discussion

Hollow heart is a common phenomenon in cucumber production that seriously affects the yield and quality. In this study, candidate genes and biological pathways associated with the formation of hollow hearts were identified, and possible regulatory patterns were predicted through transcriptome sequencing and analysis.

### Phenotype identification

Under the condition of consistent cultivation and field management, the fruits of K07 showed non-hollow or different degrees of hollow heart on a plant (Fig. 1A). In general, the phenotype of F_1_ should be consistent, and no trait separation. However, because the hollow heart was easily affected by the environment [8], the fruits at different growth stages on the same plant of K07 showed different degrees of hollow heart when the environmental conditions changed slightly (such as high temperature or continuous cloudy days). For some inbreeding lines (such as D0432-3-4), hollow hearts appeared only in extremely unsuitable environments and almost did not appear in conventional management. The molecular mechanism and how genes interact with the environment in the formation of hollow heart remain to be studied.

### Transcriptome sequencing and functional enrichment analysis of DEGs

In this study, comparative transcriptomics technology was used to explore the possible causes of the hollow heart of cucumber for the first time. Hollow and non-hollow fruits of the same variety were used (Fig. 1A), which could reduce the interference of background between different varieties and improve the accuracy of transcriptome sequencing [21]. However, transcriptome sequencing was performed only from the perspective of different parts of the fruit, and further studies may be performed from the perspective of the developmental period or other. Among the 253 DEGs identified in this study that were closely related to the formation of hollow heart, two genes reported previously, *CsALMT2* [22] and *CsG1D1a* [23], did not appear (Additional File 2). The functional enrichment analysis was not significantly enriched about the function of *CsALMT2*, but *CsG1D1a* (hormone-mediated signaling pathway and hormone metabolic process in GO, Fig. 2A), indicating that there may be multiple regulatory pathways and hormones may play important roles in the the formation of hollow heart [24].

### Auxin was related to the formation of hollow heart

Auxin is a key hormone regulating plant development, and its biosynthetic pathway has been continuously revealed [33]. Through GO enrichment analysis, the auxin synthesis pathway was significantly enriched (Fig. 2A). The genes related to IAA synthesis in gene expression pattern analysis were also significantly related to the hollow heart (Fig. 3). The pathwayof IAM or IPA is the major route to IAA in plants [34]. The expression levels of genes in the two pathways were lower in the carpel of the hollow fruit than non-hollow fruit (Fig. 3A), and the concentration of auxin in the carpel of hollow fruit was also decreased (Fig. 3B&C). The analysis proved that auxin was indeed different in the carpel and was closely associated with the formation of the hollow heart. However, there has been no report on the relationship between these key genes in the auxin biosynthesis pathways and the formation of hollow heart previously. In addition, there are some non-Trp-dependent pathways in auxin biosynthesis, but their synthesis is less [28]. Therefore, this study only analyzed the expression patterns of the main synthetic pathways. Besides, the temporal and spatial distribution of auxin is dynamically regulated by auxin metabolism, transport, and signal transduction [35]. Whether auxin is synthesized in the carpel or other parts but forms a different distribution caused by the transportation process needs to be further studied.

### Cell wall biosynthesis pathway was related to the formation of hollow heart

The plant cell wall are composed of different proportions of cellulose, hemicellulose, lignin, pectin, and some wall proteins [29]. The expression patterns of three main components (lignin, cellulose, and hemicellulose) of the cell wall were analyzed in this study [36]. The results showed that the genes in the lignin biosynthesis pathway had obvious regularity in the expression level, followed by xyloglucan and cellulose (Fig. 4). Lignin deposition around the vascular tissue and cell wall degradation in pith tissues were found to be involved in the stem cavities of Chinese flowering cabbage after harvest [37]. Wu et al. also found that the juice sac granulation was closely related to cell wall metabolism in late-ripening navel orange [17]. In cucumber, the lignin enriched in the middle of the two cell layers may result in carpel separation and cavity formation [24]. It was speculated that the blockage of lignin biosynthesis in carpel cells was one of the reasons for the formation of hollow hearts. Xyloglucan or other hemicellulose may be related to the formation of hollow heart but was not as obvious as lignin. However, *FcXTH2* (xyloglucan endotransglycosylase/hydrolases) was found to be an important enzyme acting during the biosynthesis of the cell wall and the development of the Chilean strawberry, indicating that hemicellulose may also play important roles [38]. The main xyloglucan structures present are XLFG, XXXG, and XXFG, accounting for 43%, 25%, and 24%, respectively [39]. In this study, the structure of XLFG was analyzed and other structures need to be further studied. In addition, the relationship between other components of the cell wall and hollow heart needs further study [40].

### Correlation of genes between IAA and cell wall biosynthesis

The analysis of biosynthesis pathways found that IAA and cell wall may be related to the formation of hollow heart. Pearson coefficient matrix analysis was performed to explore whether there was a correlation between these biosynthesis pathways. Pearson coefficient matrix analysis showed that there were strongly correlation between genes in IAA and cell wall biosynthesis pathways (Fig. 5). There were indeed links between auxin and cell wall biosynthesis according to previous studies. IAA can regulate the expression of cell wall relaxation factors, reshape the network structure of cell wall polysaccharides, rapidly increase the expansion of the cell wall, regulate the expression of expansin and cell wall relaxation proteins through polar transportation, and promote the expansion and growth of the cell wall [41, 42]. For example, IAA promotes lignin deposition by enhancing the biosynthesis of phenylpropanoid [43] and inhibiting *ARF2* (*auxin response factor 2*) [44]. In addition, auxin has an effect on the distribution and assembly of cellulose [45, 46]. The expression of cellulose biosynthesis-related genes was also significantly upregulated after exogenous IAA treatment [47]. From another perspective, auxin can induce the degradation of xyloglucan to elongate cells [48, 49]. IAA affected the expression of *XET* (encoding xyloglucan endotransglycosylase) and caused cell wall loosening [50]. Besides, auxin slao affects specific functional proteins to affect cell development, such as rapid alkalinization factor 1 and expansin A5 [51, 52].

### Analysis of differentially expressed TFs

TFs play an important role in transcriptional regulation. The number of TFs in cucumber is large, and the regulation patterns are also diverse. Among the 139 differentially expressed TFs identified in this study, MYB family members were the most abundant (Fig. 6). MYB is closely related to plant growth and development, secondary metabolism, and response to adversity stress [53–55]. Six MYB-related genes were annotated as xyloglucan endotransglucosylase/hydrolase 16 (Additional File 3), indicating that they may play a key role in regulating hemicellulose biosynthesis-related genes to affect cell wall formation and indirectly participate in the formation of hollow hearts [56]. Most members of NAC, ERF, and bHLH were also annotated, which are also involved in various biological processes during plant growth and development [57–60]. The two bHLH transcription factors *SPATULA* and *ALCATRAZ* reported previously [24] were also not in the 139 differentially expressed TFs identified in this study. It was speculated that hollow heart was not caused by a single transcription factor but by a series of transcription factors, forming a regulatory network coordinated and inhibited with each other [44].

## Conclusions

**Fig. 8:**
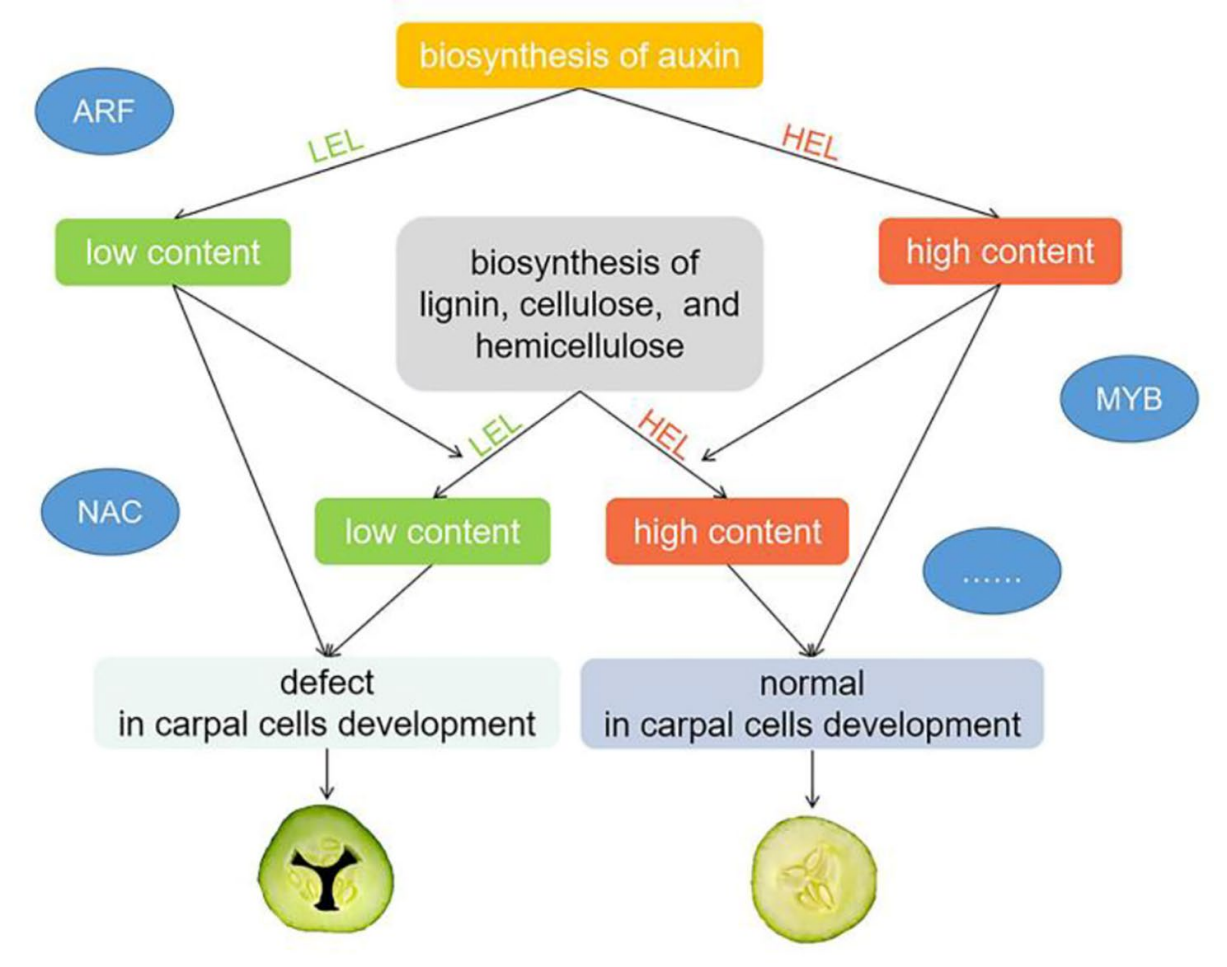
Predicted regulatory pattern in the formation of hollow heart in cucumber. The low content of auxin in the carpel affected the activity of enzymes related to cell wall biosynthesis at the early stage of fruit development, thus forming a hollow heart, some TFsmay play regulatory roles. HEL, high expression level; LEL, low expression level. The position of transcription factors in the figure was only hinted and may not be directly related to the process near them

This study predicted a new possible regulatory model for the formation of hollow heart in cucumber. The low content of auxin in the carpel affected the activity of enzymes related to cell wall biosynthesis at the early stage of fruit development, resulting in incomplete development of carpel cells, thus forming a hollow heart. Some transcription factors may play regulatory roles (Fig. 8). The results may enrich the theory of the formation of hollow heart and provide a basis for future research, and the specific gene function and actual regulatory mode need to be further studied.

## Materials and methods

### Plant growth and phenotype identification

F_1_ (K07) seeds of inbreeding lines D0432-3-4 (non-hollow) × D0708-3 (hollow) were provided by the College of Horticulture and Landscape Architecture, Northeast Agricultural University. Seeds were sown in 5 × 5 cm feeding blocks and grown in a greenhouse (30 ± 5 °C/20 ± 5 °C, 75% ± 10% relative humidity). After growing to the two-leaf stage, 50 seedlings were planted with 35 cm for plant spacing and 60 cm for row spacing in the greenhouse. Artificial pollination was carried out during the flowering period, followed by conventional field management. On the 9th day after pollination, the fruits were harvested and cut from the middle to obtain cross sections. The cross section was photoed and measured by ImageJ. The hollow degree was calculated through the formula: (area of hollow/area of cross section)×100%.

### Sample collection and total RNA extraction

Hollow fruits were identified and observed according to the standards established by Qin et al. [25]. Samples were collected on the 9th day after pollination on a plant of K07. Five grams carpel of hollow fruit (T1), 5 g flesh of hollow fruit (T2), 5 g carpel of non-hollow fruit (C1), and 5 g flesh of non-hollow fruit (C2) were collected. Each sample was subjected to three biological replicates. After collection, the samples were quickly placed into liquid nitrogen and then transferred to a refrigerator (-80 ℃). Total RNA was extracted from samples by using the EasyPure® Plant RNA Kit (TransGen Biotech, Beijing, China) by the manufacturer’s instructions. Total RNA was examined by a NanoPhotometer® spectrophotometer (IMPLEN, CA, USA). cDNA was synthesized using ReverTra Ace™ qPCR RT Master Mix with gDNA remover (TOYOBO, Osaka, Japan).

### Transcriptome sequencing and analysis

Preparation and sequencing (Illumina NovaSeq 6000) of 12 cDNA libraries were performed by ANOROAD GENOME (Beijing, China). Clean reads were obtained by removing low-quality sequences and removing joint contamination of the raw reads. All analyses in this study were based on high-quality clean data. The dataset is available in the NCBI SRA database (https://www.ncbi.nlm.nih.gov/sra/PRJNA992973). DESeq2 was used to analyze DEGs and transcription factors, with| log2 fold change| ≥ 1 and padj < 0.05. GO terms and KEGG pathways with padj < 0.05 were considered significant enrichment of DEGs. Zscore was used to represent the level of gene expression in expression profiles [61]. Corrplot of gene expression was based on the Pearson calculation method and clustered using ward. D2 [62].

### Physiological assays

Samples were collected in the middle (including fruit peel, flesh, and carpel) of hollow and non-hollow fruits on the 9th day after pollination. The concentration of soluble protein was determined according to the method of Liu et al. [63]. The concentration of soluble sugar was determined according to the method of Ding et al. [64]. Samples were collected in the middle (including fruit peel, flesh, and carpel) of hollow and non-hollow fruits on the 9th, 16th, and 23rd days after pollination, which were used to explore the changes of IAA in different developmental stages. Samples collected for transcriptome sequencing were also used to explore the changes of IAA in different parts of fruit. The concentration of IAA was determined by enzyme-linked immunosorbent assays [65] using the kit provided by China Agricultural University. Samples collected for transcriptome sequencing were used to determine the content of lignin, cellulose, and hemicellulose in fruit. The contents were determined according to the method of Aguilera-Saez et al. [66].

All determinations were carried out three times. The experimental results were expressed as the mean ± standard error and analyzed in Excel 2010 and SAS 9.4. Duncan’s test at significance levels of *p* < 0.05 and *p* < 0.01 was used to analyze the significance of the difference.

### qRT‒PCR verification

The qRT‒PCR method was performed according to Taylor et al. [67]. The samples were the same as those used for transcriptome sequencing. *Elongation factor 1α* (*Ef1α, CsaV3_2G011610*) was used as an internal reference gene. Gene-specific primers were designed (Additional File 1), and ChamQTM SYBR® qPCR Master Mix (Vazyme, Jiangsu, China) was used. The relative expression level of genes was standardized by the 2^−ΔΔCт^ method [13].

### Electronic supplementary material

Below is the link to the electronic supplementary material.

Supplementary Material 1

Supplementary Material 2

Supplementary Material 3
